# Costs associated with insufficient physical activity in Germany: cross-sectional results from the baseline examination of the German national cohort (NAKO)

**DOI:** 10.1007/s10198-024-01697-9

**Published:** 2024-05-10

**Authors:** Sophie Gottschalk, Hans-Helmut König, Andrea Weber, Michael F. Leitzmann, Michael J. Stein, Annette Peters, Claudia Flexeder, Lilian Krist, Stefan N. Willich, Katharina Nimptsch, Tobias Pischon, Sylvia Gastell, Karen Steindorf, Florian Herbolsheimer, Nina Ebert, Karin B. Michels, Anja Dorrn, Volker Harth, Nadia Obi, André Karch, Henning Teismann, Henry Völzke, Claudia Meinke-Franze, Leon Klimeck, Teresa L. Seum, Judith Dams

**Affiliations:** 1 Department of Health Economics and Health Services Research, Hamburg Center for Health Economics, University Medical Center Hamburg-Eppendorf, Martinistraße 52, 20246 Hamburg, Germany; 2 Department of Epidemiology and Preventive Medicine, University of Regensburg, Regensburg, Germany; 3 Institute of Epidemiology, Helmholtz Zentrum München – German Research Center for Environmental Health (GmbH), Neuherberg, Germany; 4 Chair of Epidemiology, Institute for Medical Information Processing, Biometry and Epidemiology, Medical Faculty, Ludwig-Maximilians-Universität München, Munich, Germany; 5 Institute and Clinic for Occupational, Social and Environmental Medicine, University Hospital, LMU Munich, Munich, Germany; 6 Institute of Social Medicine, Epidemiology and Health Economics, Charité – Universitätsmedizin Berlin, Berlin, Germany; 7 Molecular Epidemiology Research Group, Max-Delbrück-Center for Molecular Medicine in the Helmholtz Association (MDC), Berlin, Germany; 8 Max-Delbrück-Center for Molecular Medicine in the Helmholtz Association (MDC), Biobank Technology Platform, Berlin, Germany; 9 Charité - Universitätsmedizin Berlin, corporate member of Freie Universität Berlin and Humboldt-Universität zu Berlin, Berlin, Germany; 10 NAKO Study Center, German Institute of Human Nutrition Potsdam-Rehbruecke, Potsdam-Rehbruecke, Germany; 11 Division of Physical Activity, Prevention and Cancer, German Cancer Research Center (DKFZ), Heidelberg, Germany; 12 Institute for Biometrics and Epidemiology, German Diabetes Center, Leibniz Center for Diabetes Research at Heinrich Heine University, Düsseldorf, Germany; 13 Institute for Prevention and Cancer Epidemiology, Faculty of Medicine and Medical Center, University of Freiburg, Freiburg, Germany; 14 Institute for Occupational and Maritime Medicine, University Medical Center Hamburg-Eppendorf, Hamburg, Germany; 15 Institute of Epidemiology and Social Medicine, University of Münster, Münster, Germany; 16 Institute for Community Medicine, Department SHIP/ Clinical-Epidemiological Research, University Medicine Greifswald, Greifswald, Germany; 17 Division of Clinical Epidemiology and Aging Research, German Cancer Research Center (DKFZ), Heidelberg, Germany; 18 Medical Faculty Heidelberg, Heidelberg University, Heidelberg, Germany

**Keywords:** Physical activity, Exercise, Healthcare costs, Health expenditure, Cohort study

## Abstract

**Background:**

Insufficient physical activity (PA) is a leading risk factor for non-communicable diseases posing a significant economic burden to healthcare systems and societies. The study aimed to examine the differences in healthcare and indirect costs between sufficient and insufficient PA and the cost differences between PA intensity groups.

**Methods:**

The cross-sectional analysis was based on data from 157,648 participants in the baseline examination of the German National Cohort (NAKO) study. Healthcare and indirect costs were calculated based on self-reported information on health-related resource use and productivity losses. PA in the domains leisure, transport, and work was assessed by the Global Physical Activity Questionnaire and categorized into sufficient/insufficient and intensity levels (very low/low/medium/high) based on PA recommendations of the World Health Organization. Two-part models adjusted for relevant covariates were used to estimate mean costs for PA groups.

**Results and conclusion:**

Insufficiently active people had higher average annual healthcare costs (Δ €188, 95% CI [64, 311]) and healthcare plus indirect costs (Δ €482, 95% CI [262, 702]) compared to sufficiently active people. The difference was especially evident in the population aged 60 + years and when considering only leisure PA. An inverse association was observed between leisure PA and costs, whereas a direct association was found between PA at work and costs. Adjusting for the number of comorbidities reduced the differences between activity groups, but the trend persisted. The association between PA and costs differed in direction between PA domains. Future research may provide further insight into the temporal relationship between PA and costs.

**Supplementary Information:**

The online version contains supplementary material available at 10.1007/s10198-024-01697-9.

## Background

Insufficient physical activity (PA) is a global pandemic [1] and is a leading modifiable risk factor for the development of chronic, non-communicable diseases such as coronary heart disease, stroke, type 2 diabetes mellitus, and several types of cancer [2, 3]. According to the latest World Health Organization’s (WHO) guidelines on PA, substantial health benefits can be achieved if adults engage in at least 150 min per week of moderate-intensity aerobic activity (or at least 75 min of vigorous-intensity activity or an equivalent combination), plus muscle-strengthening activities twice a week [3]. Estimates for Germany suggest that less than 25% of the adult population reach this [4]. The disease burden attributable to insufficient PA has substantial economic consequences on both healthcare systems and the society as a whole [5, 6]. Hence, achieving the aim of the WHO Global Action Plan on Physical Activity [7] to reduce the global prevalence of insufficient PA by 15% by 2030 can have an important impact on population as well as individual health and well-being, healthcare systems, and the society.

In their systematic review from 2017, Ding et al. [8] summarized population-based studies examining the economic burden of insufficient PA and noted large heterogeneity in the estimates between but also within countries due to differences in methodology, measurement of PA, or cost categories considered. In general, studies using an econometric approach yielded higher estimates than those using a population-attributable fraction (PAF) approach. A PAF approach calculates the costs of insufficient PA based on the incident cases of selected key diseases/health outcomes due to insufficient PA and uses data on the costs and treatment of those specific diseases. An econometric approach, on the other hand, uses individual-level data on PA and costs (e.g., of healthcare use) and does not restrict the analysis to the costs of certain key diseases. In addition to methodological heterogeneity, the majority of studies using an econometric approach were based on older populations, an age group in which diseases related to insufficient PA typically occur [8]. Furthermore, studies examining the economic burden of insufficient PA in Germany are scarce. For example, Karl et al. used a region-specific population-based sample of people aged 48 to 68 years from Southern Germany for their cross-sectional analysis of the association between PA (self-reported sports-related and device-assessed PA) and healthcare costs [9]. They found an association of device-assessed but not self-reported insufficient PA with higher healthcare costs.

According to the WHO guidelines on PA, “there was insufficient evidence to determine whether specific health benefits vary by type or domain of physical activity”, and therefore “physical activity accrued at work, leisure, home or during transportation count towards the recommended amounts“ [3]. However, most of the previous studies on the association between PA and (healthcare) costs focused on leisure/sports-related PA or rarely asked specifically about PA in different domains (e.g [10]). In addition, the health benefits of PA are not exhausted when the threshold for “sufficient PA” is reached, but may intensify when higher levels of weekly PA are achieved [3], which may also translate into additional cost savings. Thus, analyzing costs by different PA intensity levels seems useful.

Therefore, the overall aim of the current study was to examine the costs associated with total and domain-specific insufficient PA in a large population-based sample from Germany. For this purpose, the following research questions were addressed: (1) How do healthcare and indirect costs differ between sufficiently and insufficiently active people (primary analysis) and (2) How do costs differ between different levels of PA intensity (secondary analysis).

## Methods

This manuscript was prepared in accordance with the adapted Consolidated Health Economic Evaluation Reporting Standards (CHEERS) for studies examining the economic burden of physical inactivity and other risk factors [8].

### Study design and sample

The study was a cross-sectional study based on individual-level data from the baseline examination of the German National Cohort (NAKO Gesundheitsstudie, NAKO), a multi-center, population-based prospective cohort study. Between 2014 and 2019, 205,415 persons aged 19 to 74 years participated in the baseline assessment, which consisted of a face-to-face interview, self-administered questionnaires, and several biomedical examinations [11]. Of the 204,794 participants who had not withdrawn consent by April 2023 (the time of data transfer for this study), *n* = 157,648 (78%) had complete data on the outcome (healthcare costs) and exposure (PA) of interest and represented the analysis sample of this study (*n* = 148,586 [73%] had complete data on total healthcare costs, productivity losses, and PA data).

### Outcome variable(s): healthcare and indirect costs

The primary cost perspective adopted was that of the healthcare payer. However, all analyses were also conducted from a broader perspective that additionally included certain categories of indirect costs.

Data on medication use in the last seven days (drug name, dose, and frequency of intake) was collected in the face-to-face interview. Medications were excluded if they were not prescribed by a physician (self-reported by the participants) or if they were contraceptives, homeopathic medicines, vaccines, or dietary supplements without a pharmaceutical registration number. The remaining information on health-related resource use was collected via a self-administered touchscreen questionnaire. Participants answered questions about the use and frequency/duration of outpatient general and specialist physician, inpatient, and rehabilitation services in the last 12 months. Healthcare costs were calculated by monetarily valuing resource use by standardized unit costs [12] and pharmacy retail prices [13].

Indirect costs considered in this study were costs of lost productivity due to sick leave or health-related early retirement, which were calculated using the human capital approach [14]. To this end, average gross labor costs [15] were used to monetarily value productivity losses, taking into account the extent of employment (full-/part-time) [16]. In a sensitivity analysis, costs due to productivity loss were calculated based on the friction cost approach [14], assuming a 134-day friction period [17] and a 20% replacement rate of lost productivity by other employees during the sick leave or friction period [18].

All costs were reported in 2020 euros (€) and referred to a time horizon of 12 months (in line with the period covered by the questionnaire on resource use) and were therefore not discounted.

### Exposure variable: physical activity

PA was assessed with the Global Physical Activity Questionnaire (GPAQ) [19], an extensively validated questionnaire [20–24], completed by the NAKO participants as self-administered touchscreen questionnaire. The GPAQ includes questions on the time spent in moderate and vigorous PA in a typical week in the domains leisure (sports, fitness, and recreational activities), work (paid/unpaid work, including study/training, household chores, harvesting/fishing/hunting for food, or seeking employment), and transport (the usual way to travel to and from places, excluding workplace PA) plus one question on sedentary behavior. The weekly total and domain-specific energy expenditure can be summarized by assigning metabolic equivalents of tasks (MET) to the activities. One MET is defined as the energy expenditure at rest. In line with the GPAQ analysis guide, 4 MET were assigned to moderate and 8 MET to vigorous activities [25]. The time spent in moderate/vigorous activity was multiplied by the respective MET value to obtain MET-minutes per week.

For the primary analysis, participants’ PA was classified as insufficient or sufficient according to the WHO recommendations for aerobic PA (≥ 600 MET-minutes/week, equivalent to ≥ 150 min in moderate or ≥ 75 min in vigorous PA) [3]. For the secondary analysis, PA was categorized as very low (< 40 MET-minutes/week), low (40 to < 600 MET-minutes/week), medium (600 to < 1200 MET-minutes/week), and high (≥ 1200 MET-minutes/week) [26]. The categorizations were done based on both total (all PA domains) and domain-specific PA.

### Covariates

Covariates considered for adjustment in the analyses in this study were age, sex, study site, migration background (yes; no), marital status (single or unmarried; married, cohabiting; married, not cohabiting or separated; divorced; widowed), socioeconomic status (International Socio-economic Index of Occupational Status [ISEI-08]) [27, 28], risky alcohol consumption (AUDIT-C score > 4 [male] or > 3 [female]) [29], smoking status (current smoker; former smoker; non-smoker), and the number of comorbidities (history of myocardial infarction, congestive heart failure, peripheral arterial disease, stroke, chronic lung disease, rheumatoid arthritis/polyarthritis, systemic lupus erythematodes, Sjörgren’s syndrome, gastric ulcer, liver cirrhosis, diabetes mellitus, renal insufficiency, cancer).

### Statistical analysis

Item-level missing data in covariates relevant to the analysis were low (6.3% in the socioeconomic status variable; <1% in the remaining covariates) and were replaced by single imputation techniques (e.g., mean or mode) [30]. To account for the right-skewed distribution and the zeros in the healthcare costs (9.8%) or healthcare and indirect costs (6.1%), two-part models were calculated [31]. In the first part, the probability of having non-zero costs was estimated using a probit model. In the second part, a generalized linear model (GLM) with a log-link function and gamma distribution was fitted to the non-zero values. The mean and incremental costs in/between the different PA categories (sufficient/insufficient [primary analysis] or very low/low/medium/high [secondary analysis]) were predicted from the combined first and second part models. Estimates were adjusted for covariates in two steps: Model 1 was adjusted for age, sex, study site, migration background, marital status, socioeconomic status, risky alcohol consumption, and smoking status. Model 2 was adjusted for the covariates in Model 1 plus the number of comorbidities to address potential confounding or reverse causation (e.g., individuals with low physical activity have high costs due to prior health events or pre-existing health conditions that limit their ability to be active but are also associated with high costs). Results of the primary analysis were also presented stratified by age group and separate for different cost categories.

Several additional analyses were conducted. The primary and secondary analyses were rerun when productivity losses were calculated using the friction cost approach instead of the human capital approach. The secondary analysis was repeated with PA (total and domain-specific) categorized into quintiles based on the distribution in the sample.

All analyses were conducted in Stata/MP 17.0 [StataCorp. 2021. Stata Statistical Software: Release 17. College Station, TX: StataCorp LLC]. A detailed description of the application of two-part models in Stata using the *twopm* command to analyze healthcare costs is provided by Belotti et al. [31]. All results were reported as estimated (incremental) means with corresponding 95% confidence intervals (CI) based on robust standard errors.

## Results

Sample characteristics are displayed in Table 1, for the total sample (*n* = 157,648) and separately for the sufficiently and insufficiently active groups. The mean age of the total sample was 48.7 years, 49.7% were female, and 56.9% had a high educational degree. Overall, 88% of the sample (*n* = 139,060) were classified as sufficiently active when considering PA in all three domains (leisure, work, transport). The insufficiently active group (*n* = 18,588) was more likely to be in active employment (86.9% vs. 79.5%), to have a fair to poor self-reported health status (14.1% vs. 9.4%), and to be current smokers (25.6% vs. 19.4%).

**Table 1:** Descriptive statistics of the participants of the NAKO baseline examination with valid physical activity and healthcare cost data

	Total sample (*n* = 157,648)	Sufficient PA (*n* = 139,060)	Insufficient PA (*n* = 18,588)
Age - mean (SE)	48.66 (0.03)	48.76 (0.03)	47.88 (0.08)
Female sex - n (%)	78,373 (49.71)	69,045 (49.65)	9328 (50.18)
Educational degree - n (%)			
Low	2347 (1.59)	2045 (1.57)	302 (1.74)
Medium	57,455 (38.95)	50,470 (38.79)	6985 (40.2)
High	83,930 (56.9)	74,101 (56.95)	9829 (56.57)
Still in school/vocational training	3765 (2.55)	3506 (2.69)	259 (1.49)
Employment status^a^ - n (%)			
Employed	125,880 (80.39)	109,821 (79.52)	16,059 (86.87)
Unemployed	4078 (2.6)	3622 (2.62)	456 (2.47)
Inactive	26,634 (17.01)	24,662 (17.86)	1972 (10.67)
Income (€) - mean (SE)	2357.33 (3.85)	2339.91 (4.06)	2487.83 (11.99)
Socioeconomic status (ISEI-08)^b^ - mean (SE)	51.08 (0.04)	50.92 (0.04)	52.25 (0.11)
Marital status - n (%)			
Single/unmarried	45,454 (28.84)	40,569 (29.18)	4885 (26.29)
Married, cohabiting	90,076 (57.15)	78,996 (56.82)	11,080 (59.63)
Married, not cohabiting/separated	2686 (1.7)	2356 (1.69)	330 (1.78)
Divorced	15,722 (9.98)	13,747 (9.89)	1975 (10.63)
Widowed	3673 (2.33)	3361 (2.42)	312 (1.68)
Migration background - n (%)	22,167 (14.06)	19,219 (13.82)	2948 (15.86)
Subjective general health status - n (%)			
Excellent/very good	56,533 (35.9)	51,514 (37.08)	5019 (27.03)
Good	85,455 (54.27)	74,522 (53.65)	10,933 (58.89)
Fair/poor	15,486 (9.83)	12,873 (9.27)	2613 (14.07)
Number of comorbidities^c^ - mean (SE)	0.36 (0)	0.36 (0)	0.37 (0.01)
Body mass index - mean (SE)	26.48 (0.01)	26.34 (0.01)	27.56 (0.04)
Risky alcohol consumption - n (%)	56,948 (36.14)	50,462 (36.31)	6486 (34.91)
Smoking status - n (%)			
Non-smoker, never smoked	74,257 (47.14)	66,050 (47.53)	8207 (44.18)
Previous smoker	51,567 (32.73)	45,954 (33.07)	5613 (30.22)
Smoker	31,706 (20.13)	26,951 (19.4)	4755 (25.6)

Notes: The frequencies may not add up to the total number of individuals in the group, as the frequencies and percentages were calculated based on the complete cases in the respective variables. For the subsequent analyses, missing data in the covariates considered were replaced by simple imputation techniques

^a^ Classification according to the Labour Force Concept of the International Labour Organization (ILO). ‘Inactive’ refers to all persons who are neither employed nor unemployed (e.g., people who are retired, students/below employment age, or unable to work [e.g., due to physical handicaps])

^b^ ISEI-08 (International Socio-Economic Index of occupational status) is a measure of socioeconomic status based on data on the relationship between education, occupation, and income from the International Social Survey Programme (ISSP). The score ranges from 10 to 89, with higher scores indicating higher socioeconomic status

^c^ History of myocardial infarction, congestive heart failure, peripheral arterial disease, stroke, chronic lung disease, rheumatoid arthritis/polyarthritis, systemic lupus, Sjörgren’s syndrome, gastric ulcer, liver cirrhosis, diabetes, renal insufficiency, cancer

### Primary analysis

In the primary analysis (Table 2), after adjusting for sociodemographic and behavioral risk factors (Model 1), the average total annual healthcare costs of the insufficiently active group exceeded those of the sufficiently active group by €188 (95% CI [64, 311]). The difference (Δ) was especially pronounced in the age group 60 + years (Δ €651, 95% CI [272, 1031]) and basically non-existent in the age group 40–59 years (Δ €7, 95% CI [-131, 144]). When additionally adjusting for the number of comorbidities (Model 2), the difference in healthcare costs between groups decreased but still pointed toward higher costs in the insufficiently active group (Δ €135, 95% CI [15, 255]), especially in the age group 60 + years (Δ €455, 95% CI [115, 795]). Adding indirect costs to the dependent variable, the results had a similar tendency (e.g., Δ €482, 95% CI [262, 702], total sample, Model 1), but with mean costs being considerably higher in both groups.

**Table 2:** Mean and incremental costs (2020 euros) for insufficiently vs. sufficiently active people (based on the physical activity domains leisure, transport, and work) from the NAKO baseline examination sample

		By age group		
	Total sample	20–39	40–59	60+
	Mean (95% CI)			
**Healthcare costs**				
Model 1				
Insufficient PA	2051 (1934, 2168)	1558 (1336, 1780)	1870 (1742, 1998)	3100 (2727, 3474)
Sufficient PA	1863 (1825, 1901)	1249 (1171, 1328)	1864 (1810, 1918)	2449 (2372, 2526)
Δ	188 (64, 311)	309 (82, 535)	7 (-131, 144)	651 (272, 1031)
Model 2				
Insufficient PA	2037 (1924, 2150)	1552 (1331, 1773)	1874 (1746, 2002)	2958 (2628, 3289)
Sufficient PA	1902 (1862, 1943)	1250 (1171, 1329)	1907 (1849, 1965)	2503 (2420, 2586)
Δ	135 (15, 255)	302 (77, 526)	-33 (-171, 104)	455 (115, 795)
**Healthcare + indirect costs**				
Model 1				
Insufficient PA	5419 (5210, 5627)	3166 (2885, 3448)	4646 (4411, 4882)	9843 (9066, 10,619)
Sufficient PA	4937 (4866, 5007)	2611 (2515, 2707)	4428 (4340, 4517)	8463 (8262, 8663)
Δ	482 (262, 702)	556 (264, 847)	218 (-34, 470)	1380 (575, 2185)
Model 2				
Insufficient PA	5399 (5193, 5604)	3146 (2871, 3421)	4602 (4372, 4832)	9694 (8918, 10,470)
Sufficient PA	5030 (4956, 5103)	2611 (2516, 2707)	4498 (4405, 4590)	8670 (8460, 8880)
Δ	369 (153, 586)	535 (249, 821)	104 (-141, 349)	1024 (213, 1836)

Model 1: adjusted for age, sex, study site, migration background, marital status, socioeconomic status, risky alcohol consumption, and smoking status. Model 2: adjusted for the covariates in Model 1 plus the number of comorbidities

When the classification of the sample into sufficiently and insufficiently active was based on leisure PA only, the results pointed towards higher costs in the insufficiently active group in all age groups (Table 3). In Model 1, the insufficiently active group had on average €218 (95% CI, [139, 298]) higher healthcare costs and €571 (95% CI [429, 712]) higher healthcare and indirect costs than the sufficiently active group (in Model 2: Δ €156, 95% CI, [75, 273] and Δ €416, 95% CI [272, 559], respectively).

**Table 3:** Mean and incremental costs (2020 euros) for insufficiently vs. sufficiently active people (based on leisure-time physical activity only) from the NAKO baseline examination sample

		By age group		
	Total sample	20–39	40–59	60+
	Mean (95% CI)			
**Healthcare costs**				
Model 1				
Insufficient PA	2015 (1954, 2076)	1399 (1291, 1507)	1943 (1866, 2020)	2808 (2663, 2953)
Sufficient PA	1797 (1749, 1844)	1215 (1121, 1309)	1805 (1740, 1869)	2335 (2247, 2424)
Δ	218 (139, 298)	184 (53, 314)	138 (38, 237)	473 (305, 640)
Model 2				
Insufficient PA	2010 (1948, 2072)	1387 (1281, 1494)	1941 (1862, 2021)	2742 (2601, 2882)
Sufficient PA	1854 (1804, 1904)	1221 (1125, 1317)	1872 (1802, 1941)	2426 (2330, 2522)
Δ	156 (75, 237)	166 (36, 296)	70 (-32, 171)	316 (149, 483)
**Healthcare + indirect costs**				
Model 1				
Insufficient PA	5332 (5223, 5442)	2887 (2746, 3027)	4711 (4579, 4844)	9589 (9236, 9942)
Sufficient PA	4762 (4676, 4848)	2546 (2431, 2661)	4264 (4157, 4370)	8009 (7774, 8245)
Δ	571 (429, 712)	341 (167, 514)	448 (278, 618)	1580 (1148, 2011)
Model 2				
Insufficient PA	5317 (5206, 5428)	2870 (2733, 3007)	4672 (4540, 4804)	9409 (9053, 9765)
Sufficient PA	4901 (4811, 4991)	2552 (2437, 2668)	4384 (4273, 4495)	8365 (8110, 8620)
Δ	416 (272, 559)	318 (146, 489)	288 (119, 457)	1043 (592, 1495)

Model 1: adjusted for age, sex, study site, migration background, marital status, socioeconomic status, risky alcohol consumption, and smoking status. Model 2: adjusted for the covariates in Model 1 plus the number of comorbidities

Looking at the cost differences between the PA groups by cost category (Table 4), the insufficiently active group had higher mean inpatient costs (Δ €140, 95% CI [88, 192]), outpatient costs (Δ €11, 95% CI [5, 18]), medication costs (Δ €106, 95% CI [65, 148]), indirect costs (Δ €353, 95% CI [263, 442]), and slightly lower rehabilitation costs (Δ -€14, 95% CI [-24, -3]).

**Table 4:** Mean and incremental costs (2020 euros) by cost category for insufficiently vs. sufficiently active people (based on leisure-time physical activity only) from the NAKO baseline examination sample

	Inpatient	Outpatient	Rehabilitation	Medication	Indirect costs
	Mean (95% CI)				
Model 1					
Insufficient PA	948 (905, 990)	449 (444, 454)	163 (154, 171)	471 (437, 506)	3251 (3178, 3325)
Sufficient PA	808 (776, 839)	438 (434, 442)	176 (169, 183)	365 (338, 392)	2899 (2841, 2957)
Δ	140 (88, 192)	11 (5, 18)	-14 (-24, -3)	106 (65, 148)	353 (263, 442)
Model 2					
Insufficient PA	924 (884, 965)	445 (440, 450)	159 (151, 167)	472 (436, 508)	3250 (3176, 3325)
Sufficient PA	825 (793, 857)	441 (437, 445)	179 (172, 186)	380 (351, 410)	2980 (2920, 3041)
Δ	100 (48, 151)	4 (-2, 11)	-20 (-31, -9)	92 (48, 136)	270 (180, 360)

Model 1: adjusted for age, sex, study site, migration background, marital status, socioeconomic status, risky alcohol consumption, and smoking status. Model 2: adjusted for the covariates in Model 1 plus the number of comorbidities

### Secondary analysis

**Fig. 1:**
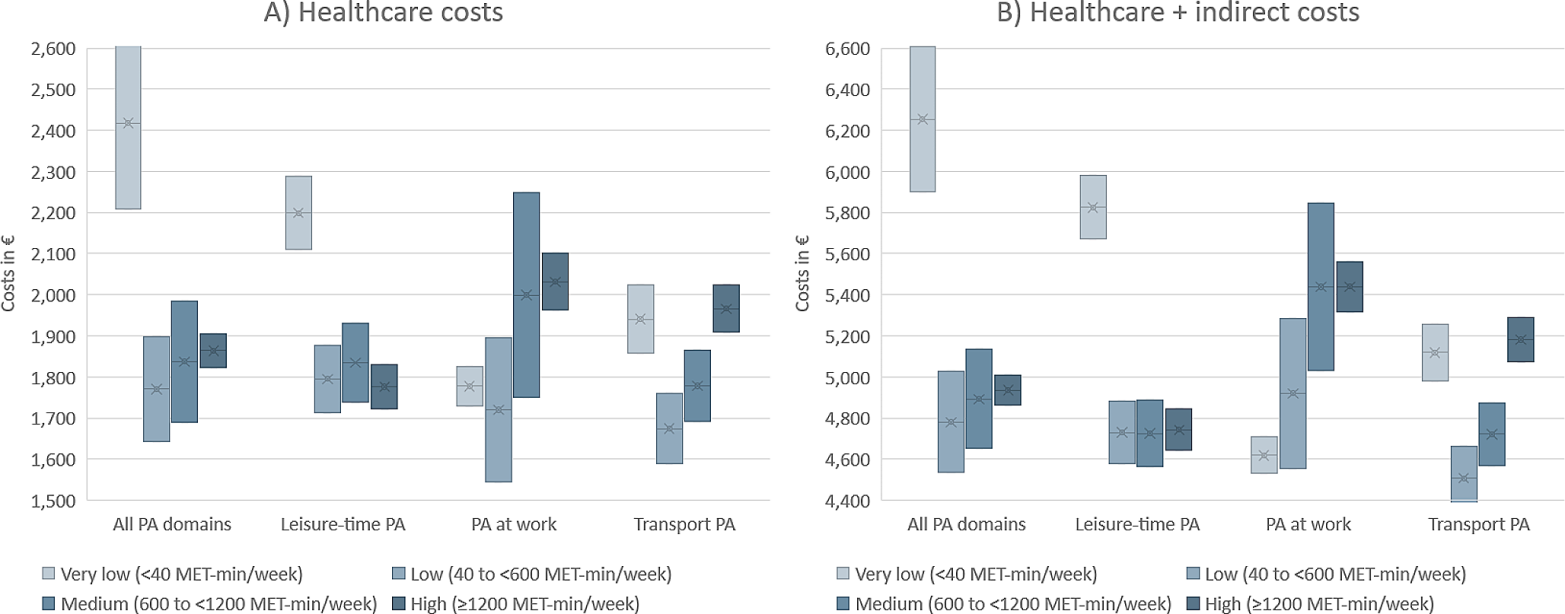
Confidence intervals of costs (2020 euros) for different physical activity levels and by activity domain of people from the NAKO baseline examination sample. Adjusted for age, sex, study site, migration background, marital status, socioeconomic status, risky alcohol consumption, and smoking status (Model 1). Crossed centerlines indicate group means

When categorized based on all PA domains, the group with very low PA showed on average higher costs than the groups with low to high PA but costs did not gradually decline with increasing PA (Table 5; Fig. 1A/B). Looking at the different domains of PA separately, the mean costs tended to be lower in the groups with low to high leisure PA compared to the group who reported very low PA in their leisure time, but increasing leisure PA from low to medium or high levels did not lead to a clear further decrease in costs. For work and transport PA, the direction of the association reversed, with the highly active group having higher mean costs than the very low PA group.

**Table 5:** Mean costs (2020 euros) for different physical activity levels and by activity domain of people from the NAKO baseline examination sample

		By PA domain		
	All PA domains	Leisure	Work	Transport
	Mean (95% CI)			
**Healthcare costs**				
Model 1				
Very low	2418 (2208, 2627)	2199 (2110, 2288)	1777 (1729, 1826)	1941 (1858, 2024)
Low	1770 (1642, 1899)	1795 (1713, 1877)	1720 (1544, 1896)	1674 (1590, 1759)
Medium	1837 (1690, 1985)	1834 (1738, 1930)	1999 (1749, 2249)	1779 (1691, 1866)
High	1864 (1824, 1904)	1776 (1721, 1831)	2031 (1962, 2100)	1966 (1909, 2023)
Model 2				
Very low	2303 (2116, 2491)	2139 (2051, 2227)	1830 (1779, 1882)	1932 (1851, 2013)
Low	1831 (1695, 1967)	1848 (1763, 1934)	1781 (1587, 1975)	1749 (1656, 1842)
Medium	1905 (1742, 2069)	1879 (1784, 1975)	2084 (1789, 2379)	1827 (1736, 1918)
High	1899 (1857, 1941)	1836 (1778, 1894)	2026 (1957, 2095)	2005 (1945, 2065)
**Healthcare + indirect costs**				
Model 1				
Very low	6254 (5901, 6607)	5825 (5670, 5980)	4619 (4530, 4707)	5119 (4979, 5258)
Low	4780 (4534, 5027)	4730 (4577, 4884)	4920 (4555, 5285)	4507 (4351, 4662)
Medium	4892 (4652, 5133)	4725 (4562, 4888)	5438 (5033, 5844)	4721 (4568, 4875)
High	4935 (4862, 5009)	4743 (4643, 4843)	5440 (5318, 5561)	5182 (5074, 5289)
Model 2				
Very low	6053 (5717, 6388)	5670 (5517, 5822)	4737 (4645, 4830)	5130 (4992, 5268)
Low	4895 (4644, 5145)	4868 (4710, 5026)	5073 (4687, 5460)	4645 (4482, 4808)
Medium	5027 (4772, 5283)	4861 (4694, 5028)	5513 (5073, 5953)	4838 (4679, 4997)
High	5022 (4946, 5099)	4882 (4778, 4987)	5449 (5327, 5572)	5269 (5157, 5380)

Model 1: adjusted for age, sex, study site, migration background, marital status, socioeconomic status, risky alcohol consumption, and smoking status. Model 2: adjusted for the covariates in Model 1 plus the number of comorbidities

### Additional analyses

Using the friction cost instead of the human capital approach for the monetary valuation of productivity losses decreased the mean costs but did not alter the results of the main analysis (Tables 7 and 8 in the appendix).

**Table 6:** Mean costs (2020 euros) for different physical activity levels (in quintiles) and by activity domain of people from the NAKO baseline examination sample

	By PA domain		
	Leisure	Work	Transport
	Mean (95% CI)		
**Healthcare costs**			
Model 1			
Q1	2200 (2111, 2289)	1779 (1730, 1827)	1940 (1857, 2023)
Q2	1807 (1727, 1888)	-	1678 (1591, 1765)
Q3	1775 (1701, 1849)	1807 (1616, 1997)	1768 (1683, 1854)
Q4	1719 (1638, 1800)	2058 (1971, 2144)	1856 (1777, 1936)
Q5	1866 (1764, 1968)	2000 (1896, 2104)	2068 (1988, 2149)
Model 2			
Q1	2140 (2052, 2228)	1831 (1780, 1883)	1930 (1850, 2011)
Q2	1857 (1773, 1940)	-	1751 (1655, 1847)
Q3	1836 (1761, 1912)	1890 (1664, 2116)	1819 (1730, 1908)
Q4	1780 (1693, 1866)	2055 (1967, 2143)	1933 (1848, 2019)
Q5	1915 (1810, 2020)	1998 (1896, 2100)	2070 (1989, 2152)
**Healthcare + indirect costs**			
Model 1			
Q1	5825 (5670, 5980)	4620 (4531, 4709)	5114 (4974, 5253)
Q2	4754 (4604, 4903)	-	4505 (4347, 4664)
Q3	4641 (4510, 4773)	5126 (4791, 5462)	4702 (4552, 4853)
Q4	4617 (4465, 4768)	5444 (5282, 5606)	4928 (4775, 5081)
Q5	4941 (4768, 5113)	5424 (5249, 5600)	5414 (5263, 5565)
Model 2			
Q1	5668 (5516, 5821)	4738 (4646, 4831)	5124 (4986, 5262)
Q2	4884 (4730, 5038)	-	4641 (4474, 4807)
Q3	4805 (4668, 4942)	5265 (4900, 5631)	4822 (4667, 4978)
Q4	4768 (4609, 4927)	5442 (5278, 5607)	5060 (4900, 5221)
Q5	5042 (4865, 5219)	5450 (5274, 5626)	5456 (5302, 5610)

Model 1: adjusted for age, sex, study site, migration background, marital status, socioeconomic status, risky alcohol consumption, and smoking status. Model 2: adjusted for the covariates in Model 1 plus the number of comorbidities

Repeating the secondary analysis with distribution-based PA intensity levels (quintiles) yielded similar results to those obtained after categorization according to predefined MET-values, but also indicated that costs decreased with higher leisure PA intensity, and only the highest PA quintile appeared to be associated with increasing costs again (Table 6; Fig. 2A/B in the appendix).

## Discussion

In this cross-sectional study based on a large population-based sample from Germany, insufficient PA was associated with higher average healthcare and indirect costs compared to sufficient PA. The difference was especially evident when considering only leisure PA and in the population aged 60 + years, an age group where chronic diseases (associated and not associated with PA) typically manifest and (healthcare) costs are generally higher. Looking at different intensity levels and domains of PA, it was found that the direction of the association between leisure PA and costs differed by PA domain, with, e.g., a non-gradual decline in costs with increasing leisure PA, whereas higher PA at work was associated with higher costs.

While the latest WHO PA guidelines state that the proposed threshold for sufficient PA may be accumulated by PA in different domains [3], there has been emerging evidence for the existence of a *PA paradox*, that is, the observation that leisure PA is health-enhancing whereas occupational PA seems to have adverse health effects, and that these effects are largely independent [32]. This PA paradox may be an appropriate explanation for the finding in the current study that people with high PA at work have higher costs than people with low PA at work. However, in the absence of a solid biological explanation for an adverse health effect of occupational PA per se, the PA paradox may be explained by the accumulation of risk factors in individuals with high occupational PA (e.g., lower socioeconomic position, behavioral and environmental risks) [33, 34]. Given the different directionality of the association between PA and costs depending on the PA domain, it seems pertinent in future studies to not simply aggregate the PA of each domain, but to study different activity patterns/profiles, taking into account (different combinations of) socioeconomic and behavioral risk factors.

The evidence collected for formulating the WHO PA guidelines further suggests that additional health benefits can be achieved by increasing PA beyond the recommended minimum (e.g., ≥ 300 min/week in moderate PA) [3]. In the current study, this was not reflected on the cost side: Low to high leisure PA was associated with lower costs compared to the group with very low PA, but no clear trend for a further decrease in costs was found in the medium and high PA groups. However, this may be related to the suspected over-reporting of PA in this study [35], where, based on leisure PA alone, 61% of the sample (more than twice the proportion of previous estimates! [4]) met the WHO recommendations for PA and > 45% were classified as highly active (> 1200 MET-min/week). The additional analysis comparing costs between PA quintiles allowed a more nuanced analysis of the association between leisure PA intensity and costs and indicated an inverse negative trend between increasing leisure PA intensity and costs that only reversed in the highest PA quintile (potentially be explained by sports-related injuries). This also suggests a non-linearity in the association between physical activity and costs, which has implications for model choices in future studies aiming, for example, to investigate the economic benefits of increasing physical activity by a certain level in a particular domain.

### Comparison with similar studies

In general, directly comparing the current study to previous (econometric) ones is difficult due to the differences in study context (e.g., country and healthcare system, population studied) and methodology (e.g., measurement of PA and costs, categorization of PA, cost components considered), which have been pointed out by previous authors [8, 36]. In a sample of U.S. non-institutionalized adults, Carlson et al. [37] found that insufficient leisure PA accounted for 8.7–11% of aggregate healthcare expenditure, and Valero-Elizondo et al. [38] found “optimal PA” (a single yes/no question about spending at least 0.5 h in moderate to vigorous PA at least five times per week) being associated with lower individual-level costs (including healthcare, out-of-pocket costs, and home health care) in people with and without cardiovascular disease and across different cardiovascular disease risk profiles. On the contrary, no association was found between leisure PA and short-term annual healthcare costs (excluding medication costs) in a sample of healthy, non-disabled adults from the U.S [39]. Similarly, in the current study, the association between PA and healthcare costs in the young to mid-aged adults was less evident when also controlling for the number of comorbidities and excluding people with severe activity impairment from the analysis. However, the difference was more pronounced and remained robust in the age group 60 + years, an age at which chronic diseases usually manifest. One of the very few longitudinal studies in the field analyzed the 12-year trajectory of PA in Australian mid-aged women and found that maintaining high leisure PA over several years was associated with the lowest healthcare and out-of-pocket costs, and that any period of increasing PA, even in later life, yielded economic benefits [40].

Only a few studies focused on different PA domains. E.g., in a relatively small sample from Brazil (*n* = 963), Codogno et al. [10] compared the percentage of people in the top healthcare expenditure quartile with the mid/high and bottom PA quartiles by PA domain (work, sport, and leisure). They found a significantly higher percentage of people from the bottom quartile of leisure PA being in the top healthcare expenditure quartile. A similar but non-significant tendency was observed for the PA domains work and sport.

In the German context, Karl et al. [9] used a regional sample to examine the direct healthcare costs of different levels of self-reported and device-based PA. They found a cross-sectional association between device-assessed but not self-reported PA and costs. However, the self-reported PA was limited to one question on sports-related PA and the time and intensity spent in PA were assessed in rather broad, pre-defined categories, whereas the GPAQ used in the NAKO study consists of several questions on the time spent in moderate and vigorous PA in each PA domain.

The current study adds to the existing evidence by using a large population-based sample from Germany to analyze the association between PA and costs, by presenting results for different PA domains, and by including not only healthcare costs but also indirect costs from sick leave or health-related early retirement, which contributed significantly to the cost differences between activity groups.

### Limitations

Several limitations need to be highlighted that led to the decision not to use the results to estimate the economic burden of insufficient PA at the population level. First, data on PA and resource use were based on self-report, which is prone to reporting bias and may have led to an underreporting of healthcare resource use or to the overreporting of PA [35, 41]. Second, the resource utilization questionnaire used in the NAKO did not ask about therapeutic or non-medical services (e.g., physical therapy) in sufficient detail to allow monetization, and the question about inpatient hospital days also did not distinguish, e.g., between intensive care, normal care, and psychiatric care. This may have led to a further underestimation of costs. Therefore, subsequent analyses that calculate healthcare costs based on claims data and objectively device-based measured PA may provide more valid results but may lack important contextual information on PA. In the NAKO, sensor-based PA was only obtained for a sub-sample and this data could not yet be requested at the time of the data usage application to the NAKO due to ongoing quality control and data preparation. Third, only productivity losses due to sick leave and health-related early retirement were considered as indirect costs, whereas PA could also affect broader societal cost categories, such as the costs of informal care or even environmental impacts (e.g., the climate-damaging effect of the healthcare sector [42]). Fourth, correction weights that account for, e.g., unequal inclusion probabilities of certain population groups are not yet available for the NAKO study, which limits the generalizability of the results to the German adult population. Finally, the cross-sectional design of the study precludes the drawing of causal inferences. Although chronic conditions are in theory assumed to be a mediator of the association between PA and costs, if preexisting conditions exist that affect a person’s ability to be physically active, they could also be a confounding factor, and thus not taking them into account could lead to an overestimation of the costs attributable to insufficient PA. For example, the difference in costs between PA levels decreased when the number of comorbidities was taken into account (although adding up existing chronic diseases to an unweighted, continuous variable could also be criticized) [8]. However, in a cross-sectional study such as this, it is difficult to disentangle the mediating and confounding effect of chronic conditions. Therefore, future longitudinal analyses (e.g. using subsequent examination waves of the NAKO) should examine the temporal association between PA and costs.

## Conclusions

In this cross-sectional study based on a large population-based sample of adults from Germany, insufficient PA was associated with higher average healthcare and indirect costs compared to sufficient PA. The association between PA intensity and costs was not gradual, and the direction of the association differed between the PA domains (e.g., an inverse association between leisure PA and costs, and a positive association between PA at work and costs). To be able to draw better conclusions about the economic effects of insufficient PA at the population level, longitudinal studies are needed that enhance understanding of the temporal relationship between PA and (healthcare) costs and the potentially mediating or confounding role of chronic diseases or health status in this context.

## Electronic Supplementary Material

Below is the link to the electronic supplementary material.

Supplementary Material 1

## Data Availability

Data of the NAKO are generally not available to the public due to strict data protection regulations. However, scientists can apply for data use according to the official usage regulation specifications. Please refer to https://transfer.nako.de for further information.
