# Supplementary material for: Costs associated with insufficient physical activity in Germany: cross-sectional results from the baseline examination of the German national cohort (NAKO)
Source: Eur J Health Econ. 2024 May 10;26(1):117–28. doi: 10.1007/s10198-024-01697-9 (PMC11743391; doi:10.1007/s10198-024-01697-9)
Supplement: Supplementary file 1 — Supplementary Material 1 [file 10198_2024_1697_MOESM1_ESM.docx]

Appendix

Table 7 Mean and incremental costs (2020 euros) for insufficiently vs. sufficiently active people (based on leisure-time physical activity only) from the NAKO baseline examination sample. Indirect costs (productivity losses) monetarily valued using the friction cost approach.

|  |  | By age group |  |  |
| --- | --- | --- | --- | --- |
|  | Total sample | 20-39 | 40-59 | 60+ |
|  | Mean (95% CI) | | | |
| **Healthcare + indirect costs** | |  |  |  |
| Model 1 |  |  |  |  |
| Insufficient PA | 4135 (4051, 4219) | 2548 (2420, 2675) | 3850 (3744, 3956) | 6526 (6293, 6759) |
| Sufficient PA | 3681 (3616, 3746) | 2246 (2140, 2352) | 3520 (3435, 3606) | 5405 (5256, 5555) |
| Δ | 454 (345, 563) | 302 (144, 460) | 330 (194, 466) | 1121 (841, 1400) |
| Model 2 |  |  |  |  |
| Insufficient PA | 4113 (4029, 4197) | 2533 (2409, 2658) | 3820 (3715, 3925) | 6396 (6167, 6625) |
| Sufficient PA | 3774 (3706, 3842) | 2252 (2145, 2359) | 3610 (3521, 3699) | 5628 (5467, 5788) |
| Δ | 339 (229, 449) | 281 (125, 438) | 210 (74, 345) | 768 (484, 1053) |
| Model 1: adjusted for age, sex, study site, migration background, marital status, socioeconomic status, risky alcohol consumption, and smoking status. Model 2: adjusted for the covariates in Model 1 plus the number of comorbidities.  Table 8 Mean costs (2020 euros) for different physical activity levels and by activity domain of people from the NAKO baseline examination sample. Indirect costs (productivity losses) monetarily valued using the friction cost approach.   \|  \| By PA domain \| \| \| \| --- \| --- \| --- \| --- \| \|  \| Leisure \| Work \| Transport \| \|  \| Mean (95% CI) \| \| \| \| **Healthcare + indirect costs** \| \| \|  \| \| Model 1 \|  \|  \|  \| \| Very low \| 4494 (4374, 4614) \| 3618 (3551, 3685) \| 4014 (3903, 4124) \| \| Low \| 3700 (3584, 3817) \| 3722 (3454, 3990) \| 3491 (3373, 3608) \| \| Medium \| 3705 (3578, 3831) \| 4132 (3814, 4451) \| 3658 (3540, 3776) \| \| High \| 3654 (3578, 3730) \| 4174 (4081, 4268) \| 3976 (3896, 4057) \| \| Model 2 \|  \|  \|  \| \| Very low \| 4367 (4250, 4484) \| 3699 (3630, 3769) \| 4007 (3900, 4115) \| \| Low \| 3794 (3674, 3913) \| 3824 (3538, 4110) \| 3594 (3470, 3718) \| \| Medium \| 3794 (3666, 3922) \| 4193 (3840, 4547) \| 3737 (3615, 3859) \| \| High \| 3747 (3668, 3826) \| 4166 (4072, 4259) \| 4027 (3945, 4110) \| \| Model 1: adjusted for age, sex, study site, migration background, marital status, socioeconomic status, risky alcohol consumption, and smoking status. Model 2: adjusted for the covariates in Model 1 plus the number of comorbidities. \| \| \| \| | | | | |


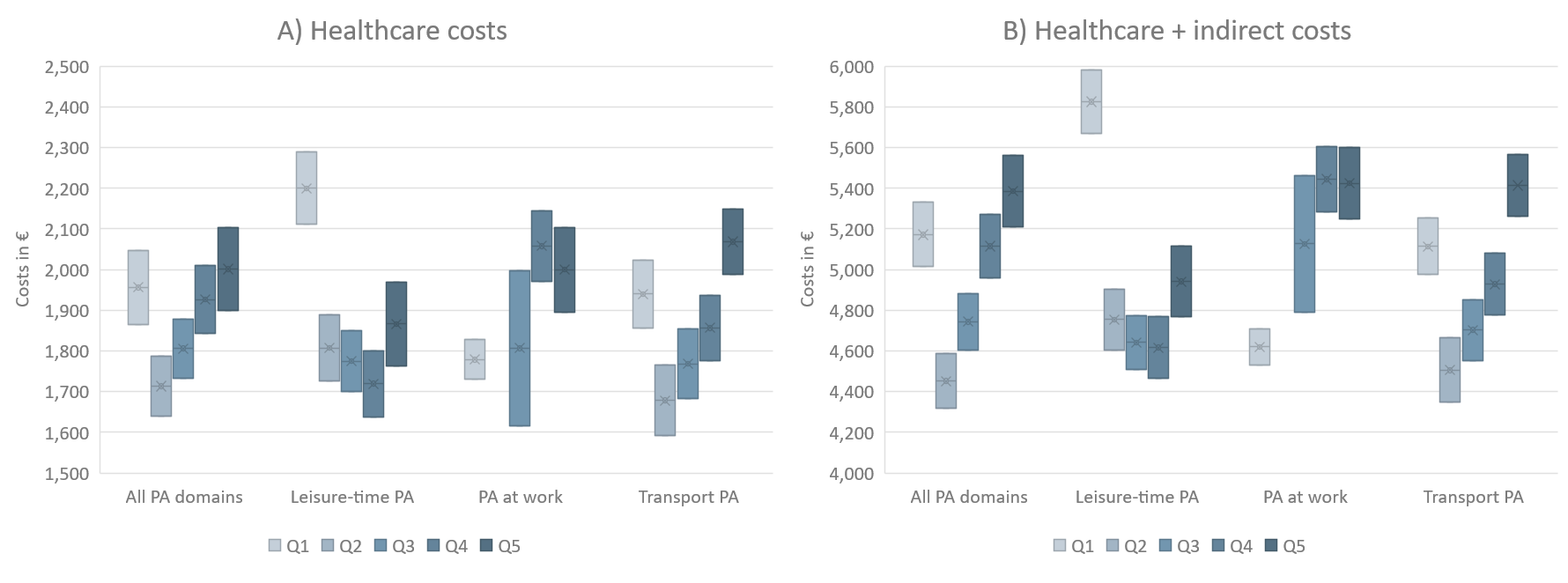


Figure 2 Confidence intervals of costs (2020 euros) for different physical activity levels (in quintiles) and by activity domain of people from the NAKO baseline examination sample. Adjusted for age, sex, study site, migration background, marital status, socioeconomic status, risky alcohol consumption, and smoking status (Model 1). Crossed centerlines indicate group means.
